# Mobile Bearing versus Fixed Bearing for Unicompartmental Arthroplasty in Monocompartmental Osteoarthritis of the Knee: A Meta-Analysis

**DOI:** 10.3390/jcm11102837

**Published:** 2022-05-17

**Authors:** Filippo Migliorini, Nicola Maffulli, Francesco Cuozzo, Karen Elsner, Frank Hildebrand, Jörg Eschweiler, Arne Driessen

**Affiliations:** 1 Department of Orthopaedic, Trauma, and Reconstructive Surgery, RWTH University Hospital, 52074 Aachen, Germany; karenelsner@netcologne.de (K.E.); fhildebrand@ukaachen.de (F.H.); joeschweiler@ukaachen.de (J.E.); adriessen@ukaachen.de (A.D.); 2 Department of Medicine, Surgery and Dentistry, University of Salerno, 84081 Baronissi, Italy; n.maffulli@qmul.ac.uk (N.M.); fra.cuoz@gmail.com (F.C.); 3 School of Pharmacy and Bioengineering, Keele University Faculty of Medicine, Stoke on Trent ST4 7QB, UK; 4 Centre for Sports and Exercise Medicine, Barts and the London School of Medicine and Dentistry, Queen Mary University of London, Mile End Hospital, London E1 4DG, UK

**Keywords:** unicompartmental knee arthroplasty, mobile bearing, fixed bearing

## Abstract

Introduction: Whether mobile-bearing (MB) unicompartmental knee arthroplasty (UKA) performs better than fixed-bearing (FB) implants in patients with monocompartmental osteoarthritis (OA) still remains unclear. Therefore, a meta-analysis comparing MB versus FB for UKA was conducted to investigate the possible advantages of MB versus FB in patient-reported outcome measures (PROMs), range of motion (ROM), and complications. We hypothesised that the MB design performs better than FB. Methods: This systematic review was conducted according to the 2020 PRISMA guidelines. In December 2021, PubMed, Web of Science, Google Scholar, and Embase were accessed, with no time constraints. All the clinical investigations comparing MB versus FB bearing for UKA were accessed. Only studies published in peer-reviewed journals were considered. Studies reporting data on revision settings were excluded, as were those combining unicompartmental and total knee arthroplasty. Results: Data from 25 studies (4696 patients) were collected; 58% (2724 of 4696 patients) were women. The mean length of follow-up was 45.8 ± 43.2. The mean age of the patients was 65.0 ± 5.6 years. No difference was found in range of motion (*p* = 0.05), Knee Scoring System (*p* = 0.9), function subscale (*p* = 0.2), and Oxford Knee Score (*p* = 0.4). No difference was found in the rate of revision (*p* = 0.2), aseptic loosening (*p* = 0.9), deep infections (*p* = 0.99), fractures (*p* = 0.6), and further extension of OA to the contralateral joint compartment (*p* = 0.2). Conclusion: The present meta-analysis failed to identify the possible superiority of the MB implants over the FB for UKA in patients with monocompartmental knee osteoarthritis. Long observational investigations are required to evaluate possible long-term complications and implant survivorship. These results should be interpreted within the limitations of the present study.

## 1. Introduction

Monocompartmental osteoarthritis (OA) of the knee is common [1]. Advanced monocompartmental knee OA impairs quality of life and participation in recreational activities [2,3]. Patients with end-stage monocompartmental OA, along with competent cruciate ligaments, varus deformity <5°, range of motion (ROM) greater than 90° without flexion contracture, and body mass index (BMI) <30 kg/m^2^, are candidates for unicompartmental knee arthroplasty (UKA) [4,5,6,7]. Mobile-bearing (MB) and fixed-bearing (FB) implants are routinely used for UKA [8,9,10]. In FB implants, the polyethylene inlay is fixed into the metal tibial plateau, allowing flexion, extension, and roll-back motion [11]. In MB implants, the polyethylene insert is mobile, allowing some degree of tibial rotation over the femur [11]. Although MB implants demonstrated faster surgical duration and greater range of motion, their superiority over FB implants still remains unclear [12,13,14,15]. Previous systematic reviews and meta-analyses, which compared the two implants, were not exhaustive, finding no clinically relevant differences in patient-reported outcome measures (PROMs), ROM, and rate of complication [8,9,16,17,18,19,20,21]. However, several clinical studies have been recently published, which have not yet been considered in any previous meta-analysis, and an update of the current evidence is required [22,23,24,25,26]. Therefore, a meta-analysis comparing MB versus FB for UKA was conducted to investigate possible advantages in PROMs, ROM, and complications.

## 2. Material and Methods

### 2.1. Eligibility Criteria

All the clinical trials comparing mobile versus fixed bearing in UKA for monocompartmental knee OA were accessed. Only studies with levels I and III of evidence, according to the Oxford Centre of Evidence-Based Medicine [27], were considered. Only studies published in peer-reviewed journals were considered. Given the authors’ language capabilities, articles in English, German, Italian, French, and Spanish were eligible. Reviews, opinions, letters, and editorials were not considered. Animal, in vitro, biomechanic, and cadaveric studies were not eligible. Studies that compared the effect of MB versus FB in experimental implants or protocols were excluded, as were those combining UKA with other interventions. Studies reporting data on revision settings were excluded, as were those combining combined results of uni- and bicompartmental arthroplasty. Only studies that clearly reported the number of patients included and the length of follow-up were eligible. Only studies that reported quantitative data under the endpoint of interest were considered for inclusion.

### 2.2. Search Strategy

This systematic review was conducted according to the Preferred Reporting Items for Systematic Reviews and Meta-Analyses: the 2020 PRISMA statement [28]. The PICOT algorithm was preliminarily pointed out:

P (Population): end-stage monocompartmental knee OA;

I (Intervention): UKA;

C (Comparison): MB versus FB;

O (Outcomes): PROMs, ROM, and complications.

In December 2021, the following databases were accessed: PubMed, Web of Science, Google Scholar, and Embase. No time constraints were used for the search. The following keywords were used in combination using the Boolean operator AND/OR: *knee, unicompartmental, unicondylar, osteoarthritis, arthroplasty, replacement, prosthesis, implant, bearing, mobile, fixed, design, range of motion, ROM, function, patient-reported outcome measures, PROMs, complications, revision, reoperation, function, quality of life, loosening, pain.*

### 2.3. Selection and Data Collection

Two authors (F.C. and K.E.) independently performed the database search. All the resulting titles were screened, and, if suitable, the abstract was accessed. The full text of the articles that matched the topic was accessed. If the full-text article was not available, the study was excluded from the present investigation. A cross-reference of the bibliography of the full-text articles was also performed. Disagreements between the authors were discussed and solved.

### 2.4. Data Items

Two authors (F.C. and K.E.) independently performed data extraction. Generalities and patient demographics of the included studies were retrieved at baseline: author and year, study design, length of follow-up, number of patients with related mean age, mean BMI, sex, Knee Scoring System (KSS) [29], and ROM. Data on ROM and on the following PROMs at last follow-up were retrieved: KSS and related function subscale (KSFS) [29] and Oxford Knee Score (OKS) [30]. Moreover, the rate of revision, deep infection, aseptic loosening, and fractures were also collected. The rate of patients who develop OA of the other knee compartment was also evaluated.

### 2.5. Study Risk of Bias Assessment

Two authors (F.C. and K.E.) independently performed the risk of bias assessment using the Review Manager (Rev.Man. 5.3, the Nordic Cochrane Collaboration, Copenhagen, Denmark). To evaluate the quality of the methodological assessment, the risk of bias graph was performed and evaluated. The following biases were evaluated: selection, detection, attrition, reporting, and others. To evaluate the overall risk of publication bias, a funnel plot of the most commonly reported outcome was performed. Asymmetries on the plot were associated with a greater risk of publication bias.

### 2.6. Synthesis Methods

All statistical analyses were performed by the first author (F.M.). For descriptive statistics, the IBM SPSS software was used. Mean and standard deviation were evaluated. For baseline comparability, the t-test was performed. Values of *p* > 0.1 indicated baseline comparability. For the meta-analyses, the Review Manager software version 5.3 (the Nordic Cochrane Collaboration, Copenhagen) was used. Continuous data were analysed using the inverse variance method and mean difference (MD) effect measure. Binary data were analysed using the Mantel–Haenszel method and the odds ratio (OR) effect measure. The comparisons were performed with a fixed model effect as set up. Heterogeneity was assessed through the χ^2^ and Higgins-I^2^ tests. If the χ^2^ < 0.05 and I^2^ tests > 50%, statistically significant moderate to high heterogeneity was detected, and a random model effect was adopted. The confidence intervals (CI) were set at 95% in all comparisons. The overall effect was considered statistically significant if *p* < 0.05. Forest and funnel plots were performed.

## 3. Results

### 3.1. Study Selection

**Figure 1:**
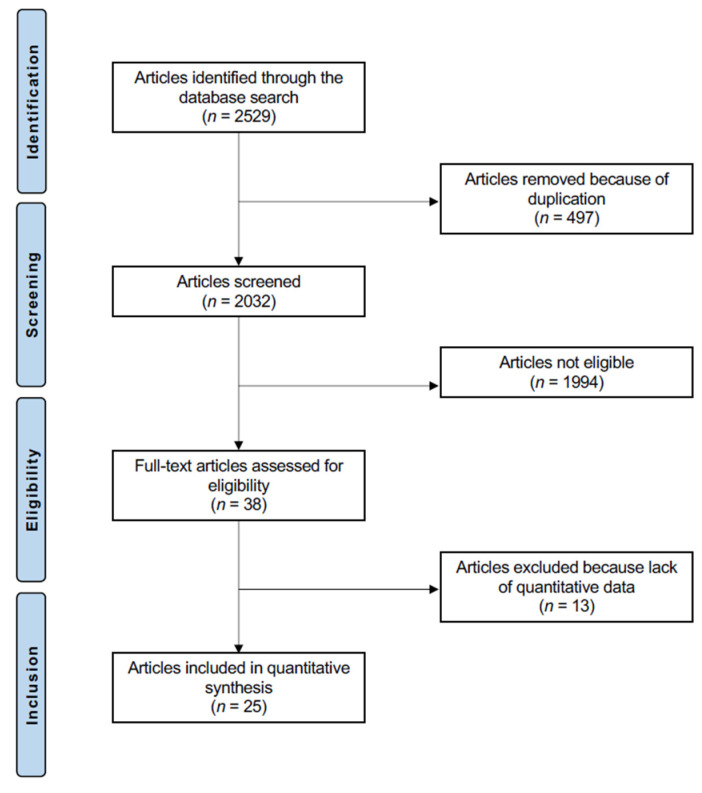
Flow chart of the literature search.

A total of 2529 papers were found in the initial literature search. Of them, 497 were excluded because of redundancy. A further 1994 articles were not eligible: not comparing mobile versus fixed bearing for UKA in a clinical setting (*n* = 1059), study type and design (*n* = 825), poor level of evidence (*n* = 31), experimental implants/protocols (*n* = 7), combining arthroplasty with other interventions (*n* = 11), combining unicompartmental and total knee arthroplasty (*n* = 4), other body regions (*n* = 37), missing information on sample size and follow-up (*n* = 8), language limitation (*n* = 9), and uncertain results (*n* = 3). Thirteen studies did not report any quantitative data under the outcome of interest and were excluded from the present investigation. Finally, 25 articles were included for analysis (Figure 1).

### 3.2. Methodological Quality Assessment

**Figure 2:**
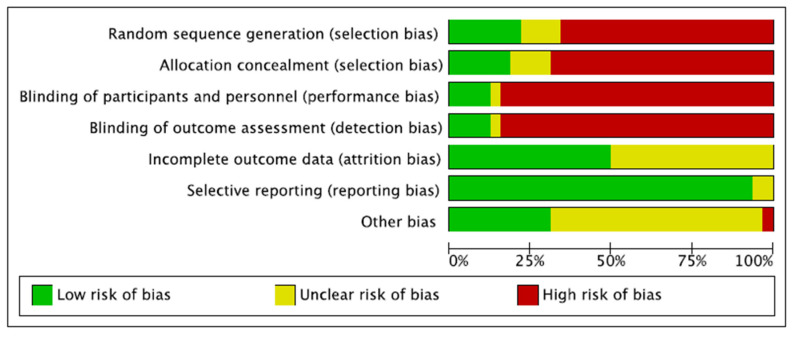
Risk of bias assessment. The risk of bias tool assessed the risk of bias (low, unclear, or high) per each risk of bias item presented as percentages across all included studies. The risk of selection bias evaluated the random sequence generation and the allocation concealment. The risk of detection bias assessed the blinding procedure during the outcome assessment. The risk of attrition bias refers to incomplete outcome data during study enrollment or analysis. The risk of reporting bias analyses the selective publication of results based on their statistical or clinical relevance. If the authors identified additional risks of bias, these were considered as “other bias”.

As only 20% (5 of 25) of the included studies performed a random allocation, the risk of selection bias was moderate to high. Performance and detection biases were also high, as assessors and patient blinding were seldom performed. Attrition and reporting biases were both low. The risk of other potential biases was moderate. Overall, the overall risk of bias was moderate (Figure 2).

### 3.3. Risk of Publication Bias

**Figure 3:**
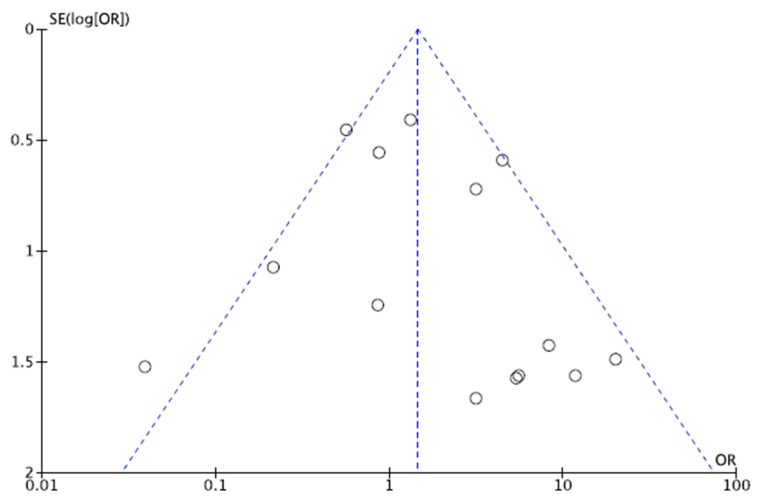
Funnel plot. The funnel plot charted the standard error (SE) of the log odds ratio (Log OR) versus its odd ratio. The degree of asymmetry of the plot is directly proportional to the degree of bias.

To assess the risk of publication bias, the funnel plot of the most commonly reported outcome (rate of revision) was performed. The plot evidenced adequate symmetry of the referral points. Egger’s test resulted in *p* = 0.3, attesting to this publication a low risk of publication bias. The funnel plot is shown in Figure 3.

### 3.4. Study Characteristics and Results of Individual Studies

Data from 4696 patients were collected; 58% (2724 of 4696 patients) were women. The mean length of follow-up was 45.8 ± 43.2 months. The mean age of the patients was 65.0 ± 5.6 years. At baseline, comparability between the MB and FB groups was found in terms of mean age and BMI, sex, mean KSS, and ROM (*p* > 0.1). Study generalities and patient demographic at baseline are shown in greater detail in Table 1.

### 3.5. Results of Syntheses

No difference was found in ROM (*p* = 0.05), KSS (*p* = 0.9), KSFS (*p* = 0.2), and OKS (*p* = 0.4). No difference was found in the rate of revision (*p* = 0.2), aseptic loosening (*p* = 0.9), deep infections (*p* = 0.99), fractures (*p* = 0.6), and further extension of OA to the contralateral joint compartment (*p* = 0.2). These results are shown in greater detail in Table 2.

## 4. Discussion

According to the main findings of the present study, MB implants performed in a similar fashion to FB implants for UKA. No difference was found in KSS, KSFS, OKS, ROM, and rate of complication.

Previous systematic reviews and meta-analyses that compared the two implants were not exhaustive, finding no clinically relevant differences in patient-reported outcome measures (PROMs), ROM, and rate of complication [8,9,16,17,18,19,20,21]. The present study updated current evidence, including recently published clinical investigations [22,23,24,25,26], investigating also additional endpoints that were not investigated by previous meta-analyses (ROM, KSFS, KOS). Ko et al. [19] included in a systematic review 1019 procedures (10 studies), finding a similar rate of complication between the two implants. Similar results were evidenced by Peersman et al. [8] in a systematic review of 9463 knees (44 studies). Cheng et al. [20] performed a meta-analysis involving 915 knees (nine studies). The authors found no difference in clinical outcomes and complication rates. Zhang et al. [9], in a recent meta-analysis involving 2612 procedures (14 studies), reported no difference in KSS, OKS, ROM, and complications. On the contrary, Burger et al. [21], in a systematic review including 2265 procedures (28 studies), concluded that MB reported a greater rate of revision compared to FB implants and similar clinical outcomes. The present study evidenced no difference between the two implant designs in the rate of revision, aseptic loosening, and OA progression. These results were confirmed by previous similar meta-analyses [16,17,18]. The most common reasons for revision following UKA implantation are aseptic loosening, progression of arthritis, and wear of the polyethylene insert [7,16,17,19]. Given their more congruent bearing surfaces with a larger contact area, MB implants have been introduced to reproduce better anatomic knee motion, minimise constraints, contact stress, and, thus, polyethylene wear [35,47,48,49]. These features should reduce implant loosening and polyethylene wear and favour longer MB implant survivorship [50,51]. However, suboptimal implant alignment and soft-tissue balancing can lead to bearing dislocation or impingement [37]. Indeed, MB implants are very sensitive to soft-tissue balancing [19]. Any undercorrection of the articular compartment promotes higher component stress contributing to polyethylene dislocation. On the other hand, any overcorrection promotes greater contact stress in the contralateral compartment, accelerating OA progression [4]. Given its flat tibial articular surface, FB implants are easier to implant, and the risk of bearing dislocation is minimal [35,49,52]. In this respect, FB implants could offload the contralateral compartment, slowing or preventing osteoarthritis progression [19]. The flat tibial component of FB implants, given their fatigue and shear-stress-related mechanism, are less compliant during flexion and can lead to point loading; hence, they are more prone to inlay surface deformation and delamination [53,54]. However, the results from the present study did not evidence any difference in the rate of OA progression between the two implants.

The present study certainly has limitations. The retrospective design of most of the included studies is an important limitation. Indeed, only 5 of 25 included studies performed randomised allocation, which represents an important source of selection bias. The limited length of the follow-up in many included studies represents another important limitation, which limits the reliability of the present investigation and jeopardises the ability to identify possible longer-term complications. The current literature lacks long-term randomised controlled trials, and future high-quality investigations are required. The postoperative rehabilitation protocol was seldom described, and the general health information of the included patients is often missing. Although the description of the surgical technique was adequately reported in most studies, the surgeon’s experience was barely stated. The latter may influence the clinical outcome, especially in MB implants, which require a longer learning curve and accurate soft-tissue balancing [55,56,57]; however, given the limited available data for inclusion, it was not possible to consider this endpoint for analysis. Given the lack of quantitative data, the analyses were conducted regardless of the type of the implant. Heterogeneities were found with regard to the implant manufacturers. Other studies did not specify which implant they used or combined two or more implants. Given these heterogeneities, no further analyses were possible to conduct. Given these limitations, results from the present study should be considered cautiously.

## 5. Conclusions

The present meta-analysis failed to identify the possible superiority of the MB implants over the FB for UKA in patients with monocompartmental knee osteoarthritis. Long observational investigations are required to evaluate possible long-term complications and implant survivorship. These results should be interpreted within the limitations of the present study.

## Figures and Tables

**Table 1:** Generalities and patient baseline of the included studies (MB: mobile bearing; FB: fixed bearing).

Author, Year	Journal	Design	Follow-Up (Months)	Bearing	Procedures (*n*)	Mean Age	Women (*%*)
Artz et al., 2015 [31]	*J. Arthroplasty*	Randomised	24	MB	205	62.0	50%
				FB	284	71.4	44%
Bhattacharya et al., 2012 [32]	*Knee*	Retrospective	44.7	FB	91	67.7	58%
				MB	49	68.8	47%
Biau et al., 2013 [33]	*J. Arthroplasty*	Retrospective	24	MB	33	67.7	59%
				FB	57	68.8	51%
Catani et al., 2011 [34]	*Knee Surg Sports Traumatol Arthrosc*	Retrospective	12	MB	10	70.3	80%
				FB	10	70.3	60%
Confalonieri et al., 2004 [13]	*Knee*	Randomised	68.4	MB	20	71.0	45%
				FB	20	69.5	60%
Emerson et al., 2002 [35]	*Clin. Orthop. Relat. Res.*	Prospective	81.6	MB	50	63.0	56%
				FB	51	63.0	66%
Forster et al., 2007 [36]	*Knee Surg. Sports Traumatol. Arthrosc.*	Prospective	24	FB	17	75.0	69%
				MB	13	55.0	42%
Gilmour et al., 2018 [23]	*J. Arthroplasty*	Prospective	24	FB	58	61.8	45%
				MB	54	62.6	45%
Gleeson et al., 2004 [37]	*Knee*	Randomised	24	FB	57	66.7	41%
				MB	47	64.7	60%
Inoue et al., 2016 [38]	*J. Arthroplasty*	Retrospective	27.3	FB	24	75.0	76%
				MB	28	73.3	76%
Kayani et al., 2019 [24]	*Bone Joint J.*	Prospective	3	MB	73	66.1	53%
				FB	73	65.3	56%
Kazarian et al., 2020 [25]	*J. Bone Joint Surg.*	Retrospective	44.4	FB	162	63.2	59%
				MB	91	62.2	52%
Kim et al., 2016 [39]	*Knee Surg. Sports Traumatol. Arthrosc.*	Retrospective	94	MB	1441	62.0 62.0	91% 91%
				FB	135		
Kim et al., 2020 [26]	*Int. Orthop.*	Retrospective	60	FB	58	61.3	93%
				MB	57	60.7	84%
Koppens et al., 2019 [15]	*Acta Orthop.*	Randomised	24	MB	33	64.0	52%
				FB	32	61.0	47%
Li et al., 2006 [40]	*Knee*	Randomised	24	FB	28	70.0	32%
				MB	28	74.0	29%
Neufeld et al., 2018 [18]	*J Arthroplasty*	Retrospective	120	MB	38	60.3	58%
				FB	68	64.6	50%
Ozcan et al., 2018 [41]	*Arch. Orthop. Trauma Surg.*	Retrospective	28.8	FB	153		
				MB	171		
Paratte et al., 2011 [42]	*Clin. Orthop. Relat. Res.*	Retrospective	180	FB	79	62.8	63%
				MB	77	63.4	68%
Patrick et al., 2020 [14]	*J. Orthop. Surg. Res.*	Retrospective	14.4	MB	150	68.6	53%
				FB	44	67.7	86%
Pronk et al., 2020 [43]	*Knee Surg. Sports Traumatol. Arthrosc.*	Retrospective	12	MB	66	61.4	47%
				FB	97	61.2	44%
Seo et al., 2019 [44]	*Arch. Ortho.p Trauma Surg.*	Retrospective	120	MB	36	64.5	97%
				FB	60	61.8	95%
Tecame et al., 2018 [45]	*Int. Orthop.*	Retrospective	42	MB	9	47.8	17%
				FB	15	48.4	
Verdini et al., 2017 [46]	*Muscles Ligaments Tendons J.*	Prospective	20	MB	7	68.0	60%
				FB	8	67.0	40%
Whittaker et al., 2010 [16]	*Clin. Orthop. Relat. Res.*	Retrospective	3.6	FB	150	68.0	53%
				MB	79	63.0	48%

**Table 2:** Main results of the meta-analyses. The final effect was evaluated as odds ratio for binary data and as mean difference for continuous data (MB: mobile bearing; FB: fixed bearing; CI: confidence interval).

Endpoint	MB	FB	Model	95% CI	Final Effect	*p*	I^2^ (*%*)
ROM	243	249	Fixed	−4.37, −0.04	−2.21	0.05	0
KSS	487	548	Random	−6.38, 5.64	−0.37	0.9	99
KSFS	176	241	Fixed	−1.92, 0.31	−0.81	0.2	0
OKS	97	95	Random	−11.56, 4.44	−3.56	0.4	95
Revision	2353	1148	Random	0.82, 3.20	1.62	0.2	52
Aseptic Loosening	1810	658	Random	0.16, 7.96	1.12	0.9	89
Deep Infections	1781	404	Fixed	0.28, 3.47	0.99	0.99	0
Fractures	1679	277	Random	0.08, 4.85	0.61	0.6	62
OA Progression	1752	602	Fixed	0.81, 2.60	1.45	0.2	3

## Data Availability

The datasets generated during and/or analysed during the current study are available throughout the manuscript.

## References

[1] Oliveria S.A., Felson D.T., Reed J.I., Cirillo P.A., Walker A.M. (1995). Incidence of symptomatic hand, hip and knee osteoarthritis among patients in a health maintenance organisation. Arthritis Rheum..

[2] Tille E., Beyer F., Auerbach K., Tinius M., Lutzner J. (2021). Better short-term function after unicompartmental compared to total knee arthroplasty. BMC Musculoskelet. Disord..

[3] Panzram B., Bertlich I., Reiner T., Walker T., Hagmann S., Gotterbarm T. (2018). Cementless unicompartmental knee replacement allows early return to normal activity. BMC Musculoskelet. Disord..

[4] Kozinn S.C., Scott R. (1989). Unicondylar knee arthroplasty. J. Bone Jt. Surg. Am..

[5] Leiss F., Gotz J.S., Maderbacher G., Zeman F., Meissner W., Grifka J., Benditz A., Greimel F. (2020). Pain management of unicompartmental (UKA) vs. total knee arthroplasty (TKA) based on a matched pair analysis of 4144 cases. Sci. Rep..

[6] Argenson J.N. (2017). Unicompartmental knee arthroplasty versus total knee arthroplasty. Knee Surg. Sports Traumatol. Arthrosc..

[7] Migliorini F., Tingart M., Niewiera M., Rath B., Eschweiler J. (2019). Unicompartmental versus total knee arthroplasty for knee osteoarthritis. Eur. J. Orthop. Surg. Traumatol..

[8] Peersman G., Stuyts B., Vandenlangenbergh T., Cartier P., Fennema P. (2015). Fixed-versus mobile-bearing UKA: A systematic review and meta-analysis. Knee Surg. Sports Traumatol. Arthrosc..

[9] Zhang W., Wang J., Li H., Wang W., George D.M., Huang T. (2020). Fixed- versus mobile-bearing unicompartmental knee arthroplasty: A meta-analysis. Sci. Rep..

[10] Capella M., Dolfin M., Saccia F. (2016). Mobile bearing and fixed bearing total knee arthroplasty. Ann. Transl. Med..

[11] Schneider D.T., Ostermeier P.S., Rinio D.M., Marquaß P.D.B. Fixed vs Mobile Bearing Prothesis. https://www.joint-surgeon.com/orthopedic-services/knee/total-replacement-knee-types-of-procedures.

[12] Huang F., Wu D., Chang J., Zhang C., Qin K., Liao F., Yin Z. (2021). A Comparison of Mobile- and Fixed-Bearing Unicompartmental Knee Arthroplasties in the Treatment of Medial Knee Osteoarthritis: A Systematic Review and Meta-analysis of 1861 Patients. J. Knee Surg..

[13] Confalonieri N., Manzotti A., Pullen C. (2004). Comparison of a mobile with a fixed tibial bearing unicompartimental knee prosthesis: A prospective randomized trial using a dedicated outcome score. Knee.

[14] Ng J.P., Fan J.C.H., Lau L.C.M., Tse T.T.S., Wan S.Y.C., Hung Y.W. (2020). Can accuracy of component alignment be improved with Oxford UKA Microplasty(R) instrumentation?. J. Orthop. Surg. Res..

[15] Koppens D., Rytter S., Munk S., Dalsgaard J., Sorensen O.G., Hansen T.B., Stilling M. (2019). Equal tibial component fixation of a mobile-bearing and fixed-bearing medial unicompartmental knee arthroplasty: A randomized controlled RSA study with 2-year follow-up. Acta Orthop..

[16] Whittaker J.P., Naudie D.D., McAuley J.P., McCalden R.W., MacDonald S.J., Bourne R.B. (2010). Does bearing design influence midterm survivorship of unicompartmental arthroplasty?. Clin. Orthop. Relat. Res..

[17] Bruce D.J., Hassaballa M., Robinson J.R., Porteous A.J., Murray J.R., Newman J.H. (2020). Minimum 10-year outcomes of a fixed bearing all-polyethylene unicompartmental knee arthroplasty used to treat medial osteoarthritis. Knee.

[18] Neufeld M.E., Albers A., Greidanus N.V., Garbuz D.S., Masri B.A. (2018). A Comparison of Mobile and Fixed-Bearing Unicompartmental Knee Arthroplasty at a Minimum 10-Year Follow-up. J. Arthroplast..

[19] Ko Y.B., Gujarathi M.R., Oh K.J. (2015). Outcome of Unicompartmental Knee Arthroplasty: A Systematic Review of Comparative Studies between Fixed and Mobile Bearings Focusing on Complications. Knee Surg. Relat. Res..

[20] Cheng T., Chen D., Zhu C., Pan X., Mao X., Guo Y., Zhang X. (2013). Fixed- versus mobile-bearing unicondylar knee arthroplasty: Are failure modes different?. Knee Surg. Sports Traumatol. Arthrosc..

[21] Burger J.A., Kleeblad L.J., Sierevelt I.N., Horstmann W.G., Nolte P.A. (2019). Bearing design influences short- to mid-term survivorship, but not functional outcomes following lateral unicompartmental knee arthroplasty: A systematic review. Knee Surg. Sports Traumatol. Arthrosc..

[22] Deckard E.R., Jansen K., Ziemba-Davis M., Sonn K.A., Meneghini R.M. (2020). Does Patellofemoral Disease Affect Outcomes in Contemporary Medial Fixed-Bearing Unicompartmental Knee Arthroplasty?. J. Arthroplast..

[23] Gilmour A., MacLean A., Rowe P., Banger M., Donnelly I., Jones B., Blyth M. (2018). Robotic-Arm Assisted Versus Conventional Unicompartmental Knee Arthroplasty. The 2 year Results of a Randomised Controlled Trial. J. Arthroplast..

[24] Kayani B., Konan S., Tahmassebi J., Rowan F.E., Haddad F.S. (2019). An assessment of early functional rehabilitation and hospital discharge in conventional versus robotic-arm assisted unicompartmental knee arthroplasty: A prospective cohort study. Bone Jt. J..

[25] Kazarian G.S., Barrack T.N., Okafor L., Barrack R.L., Nunley R.M., Lawrie C.M. (2020). High Prevalence of Radiographic Outliers and Revisions with Unicompartmental Knee Arthroplasty. J. Bone Jt. Surg. Am..

[26] Kim M.S., Koh I.J., Kim C.K., Choi K.Y., Baek J.W., In Y. (2020). Comparison of implant position and joint awareness between fixed- and mobile-bearing unicompartmental knee arthroplasty: A minimum of five year follow-up study. Int. Orthop..

[27] Howick J.C.I., Glasziou P., Greenhalgh T., Carl Heneghan Liberati A., Moschetti I., Phillips B., Thornton H., Goddard O., Hodgkinson M. (2011). The 2011 Oxford CEBM Levels of Evidence. Oxford Centre for Evidence-Based Medicine. https://www.cebm.net/index.aspx?o=5653.

[28] Page M.J., McKenzie J.E., Bossuyt P.M., Boutron I., Hoffmann T.C., Mulrow C.D., Shamseer L., Tetzlaff J.M., Akl E.A., Brennan S.E. (2021). The PRISMA 2020 statement: An updated guideline for reporting systematic reviews. BMJ.

[29] Insall J.N., Dorr L.D., Scott R.D., Scott W.N. (1989). Rationale of the Knee Society clinical rating system. Clin. Orthop. Relat. Res..

[30] Murray D.W., Fitzpatrick R., Rogers K., Pandit H., Beard D.J., Carr A.J., Dawson J. (2007). The use of the Oxford hip and knee scores. J. Bone Jt. Surg. Br..

[31] Artz N.J., Hassaballa M.A., Robinson J.R., Newman J.H., Porteous A.J., Murray J.R. (2015). Patient Reported Kneeling Ability in Fixed and Mobile Bearing Knee Arthroplasty. J. Arthroplast..

[32] Bhattacharya R., Scott C.E., Morris H.E., Wade F., Nutton R.W. (2012). Survivorship and patient satisfaction of a fixed bearing unicompartmental knee arthroplasty incorporating an all-polyethylene tibial component. Knee.

[33] Biau D.J., Greidanus N.V., Garbuz D.S., Masri B.A. (2013). No difference in quality-of-life outcomes after mobile and fixed-bearing medial unicompartmental knee replacement. J. Arthroplast..

[34] Catani F., Benedetti M.G., Bianchi L., Marchionni V., Giannini S., Leardini A. (2012). Muscle activity around the knee and gait performance in unicompartmental knee arthroplasty patients: A comparative study on fixed- and mobile-bearing designs. Knee Surg. Sports Traumatol. Arthrosc..

[35] Emerson R.H., Hansborough T., Reitman R.D., Rosenfeldt W., Higgins L.L. (2002). Comparison of a mobile with a fixed-bearing unicompartmental knee implant. Clin. Orthop. Relat. Res..

[36] Forster M.C., Bauze A.J., Keene G.C. (2007). Lateral unicompartmental knee replacement: Fixed or mobile bearing?. Knee Surg. Sports Traumatol. Arthrosc..

[37] Gleeson R.E., Evans R., Ackroyd C.E., Webb J., Newman J.H. (2004). Fixed or mobile bearing unicompartmental knee replacement? A comparative cohort study. Knee.

[38] Inoue A., Arai Y., Nakagawa S., Inoue H., Yamazoe S., Kubo T. (2016). Comparison of Alignment Correction Angles Between Fixed-Bearing and Mobile-Bearing UKA. J. Arthroplast..

[39] Kim K.T., Lee S., Lee J.I., Kim J.W. (2016). Analysis and Treatment of Complications after Unicompartmental Knee Arthroplasty. Knee Surg. Relat. Res..

[40] Li M.G., Yao F., Joss B., Ioppolo J., Nivbrant B., Wood D. (2006). Mobile vs. fixed bearing unicondylar knee arthroplasty: A randomized study on short term clinical outcomes and knee kinematics. Knee.

[41] Ozcan C., Simsek M.E., Tahta M., Akkaya M., Gursoy S., Bozkurt M. (2018). Fixed-bearing unicompartmental knee arthroplasty tolerates higher variance in tibial implant rotation than mobile-bearing designs. Arch. Orthop. Trauma Surg..

[42] Parratte S., Pauly V., Aubaniac J.M., Argenson J.N. (2012). No long-term difference between fixed and mobile medial unicompartmental arthroplasty. Clin. Orthop. Relat. Res..

[43] Pronk Y., Paters A.A.M., Brinkman J.M. (2021). No difference in patient satisfaction after mobile bearing or fixed bearing medial unicompartmental knee arthroplasty. Knee Surg. Sports Traumatol. Arthrosc..

[44] Seo S.S., Kim C.W., Lee C.R., Kwon Y.U., Oh M., Kim O.G., Kim C.K. (2019). Long-term outcomes of unicompartmental knee arthroplasty in patients requiring high flexion: An average 10-year follow-up study. Arch. Orthop. Trauma Surg..

[45] Tecame A., Savica R., Rosa M.A., Adravanti P. (2019). Anterior cruciate ligament reconstruction in association with medial unicompartmental knee replacement: A retrospective study comparing clinical and radiological outcomes of two different implant design. Int. Orthop..

[46] Verdini F., Zara C., Leo T., Mengarelli A., Cardarelli S., Innocenti B. (2017). Assessment of patient functional performance in different knee arthroplasty designs during unconstrained squat. Muscle Ligaments Tendons J..

[47] Smith T.O., Hing C.B., Davies L., Donell S.T. (2009). Fixed versus mobile bearing unicompartmental knee replacement: A meta-analysis. Orthop. Traumatol. Surg. Res..

[48] Ammarullah M.I., Afif I.Y., Maula M.I., Winarni T.I., Tauviqirrahman M., Akbar I., Basri H., van der Heide E., Jamari J. (2021). Tresca Stress Simulation of Metal-on-Metal Total Hip Arthroplasty during Normal Walking Activity. Materials.

[49] Jamari J., Ammarullah M.I., Saad A.P.M., Syahrom A., Uddin M., van der Heide E., Basri H. (2021). The Effect of Bottom Profile Dimples on the Femoral Head on Wear in Metal-on-Metal Total Hip Arthroplasty. J. Funct. Biomater..

[50] O’Connor J.J., Goodfellow J.W. (1996). Theory and practice of meniscal knee replacement: Designing against wear. Proc. Inst. Mech. Eng. Part H.

[51] Kendrick B.J., Longino D., Pandit H., Svard U., Gill H.S., Dodd C.A., Murray D.W., Price A.J. (2010). Polyethylene wear in Oxford unicompartmental knee replacement: A retrieval study of 47 bearings. J. Bone Jt. Surg. Br..

[52] Brockett C.L., Jennings L.M., Fisher J. (2011). The wear of fixed and mobile bearing unicompartmental knee replacements. Proc. Inst. Mech. Eng. Part H.

[53] Argenson J.N., Parratte S. (2006). The unicompartmental knee: Design and technical considerations in minimizing wear. Clin. Orthop. Relat. Res..

[54] Manson T.T., Kelly N.H., Lipman J.D., Wright T.M., Westrich G.H. (2010). Unicondylar knee retrieval analysis. J. Arthroplast..

[55] Zambianchi F., Digennaro V., Giorgini A., Grandi G., Fiacchi F., Mugnai R., Catani F. (2015). Surgeon’s experience influences UKA survivorship: A comparative study between all-poly and metal back designs. Knee Surg. Sports Traumatol. Arthrosc..

[56] Ridgeway S.R., McAuley J.P., Ammeen D.J., Engh G.A. (2002). The effect of alignment of the knee on the outcome of unicompartmental knee replacement. J. Bone Jt. Surg. Br..

[57] Robertsson O., Knutson K., Lewold S., Lidgren L. (2001). The routine of surgical management reduces failure after unicompartmental knee arthroplasty. J. Bone Jt. Surg. Br..

